# Post-vaccination SARS-CoV-2 infections among healthcare workers in a tertiary hospital in Ghana

**DOI:** 10.1371/journal.pone.0331971

**Published:** 2025-09-17

**Authors:** Kissinger Marfoh, Ali Samba, Eunice Okyere, Ahmad Zaid Fattah, Dorothy Naa Ashokor Darko, Prince Nuertey Odoom, Michael Darko Ashaley

**Affiliations:** 1 College of Medicine, Nursing and Health Science, Fiji National University, Suva, Fiji; 2 Public Health Department, Korle-Bu Teaching Hospital, Accra, Ghana; 3 Faculty of Medicine and Health Science, University of Putra Malaysia, Serdang, Selangor, Malaysia; 4 Obstetrics and Gynecology Department, Korle-Teaching Hospital, Accra, Ghana; 5 Medical Microbiology Department, University of Ghana, Legon, Ghana; Chulalongkorn University Faculty of Medicine, THAILAND

## Abstract

**Introduction:**

Vaccines remain the most effective preventive measure against the ever-changing severe acute respiratory syndrome coronavirus 2 (SARS-CoV-2) virus. However, vaccine access remains unequal, leaving healthcare workers in low- and middle-income countries (LMICs) like Ghana at increased risk, despite early prioritisation. These inequities threaten both individual safety and the resilience of health systems. Moreover, SARS-CoV-2 infections continue to occur, particularly with emerging variants, compounding these risks. This study aimed to investigate the incidence and risk factors associated with post-vaccination SARS-CoV-2 infections among healthcare workers at a tertiary hospital in Ghana following the administration of the ChAdOx1nCoV-19 vaccine.

**Method:**

We conducted a prospective cohort study of 4252 healthcare workers aged 18 and above, who tested negative for the SARS-CoV-2, and partially or fully vaccinated with the ChAdOx1nCoV-19 vaccine at baseline. After completing the baseline questionnaire, healthcare workers were followed up for one year.

**Results:**

2283 out of the 4252 (53.7%) healthcare workers had post-vaccination infections, with an overall incidence of 95.7 cases per 100 person-years (95% CI: 91.8–99.7) of follow-up. The incidence of breakthrough infection was 82.0 cases per 100 person-years (95% Cl 78.0–86.0). In a multivariable Cox regression, age, vaccination status, occupation, clinical stations, frontline status and previous SARS-CoV-2 infections were significantly associated with post-vaccination infections. Compared to non-clinical healthcare workers, nurses (HR = 1.91, 95% CI: 1.69–2.17) and doctors (HR = 1.37, 95% CI: 1.24–1.73) had a higher risk of post-vaccination infections. Similarly, elderly individuals (HR = 1.04, 95% CI: 1.02–1.05) and those with comorbidities (HR = 1.86, 95% CI: 1.67–1.73) were more likely to develop post-vaccination infections. Frontline healthcare workers and healthcare workers stationed at the point-of-entry services (emergency and outpatient clinics) had a high rate of infections. However, previous SARS-CoV-2 infections (HR = 0.80, 95% CI: 0.71–0.53) and full vaccination (HR = 0.56, 95% CI: 0.51–0.62) conferred some protection, despite an overall rise in infection post vaccination incidence.

**Conclusion:**

In conclusion, the results of our study suggest a high incidence of post-vaccination infections among healthcare workers in the context of varying epidemic waves. Additionally, the study identified partial or incomplete vaccination, elderly workers, comorbidities, frontline workers, nurses and point-of-entry service roles as high-risk factors for post-vaccination infections. These findings reinforce the need for tailored booster strategies and strengthened protection for high-risk healthcare workers in LMIC settings.

## Introduction

Vaccines remain the most effective preventive measure against SARS-CoV-2, and their rapid development and global rollout marked a major public health success. However, the benefits of vaccination have not been equally distributed. Stark disparities in vaccine access remain a major challenge in low- and middle-income countries (LMICs) such as Ghana, where healthcare workers remain at elevated risk of infection despite early prioritisation in vaccine rollout. These inequities threaten not only individual safety but also the resilience of already strained health systems [1,2].

Various factors have contributed to these disparities, including vaccine nationalism, supply chain disruptions, limited manufacturing capacity, and the underperformance of global allocation mechanisms such as COVID-19 Vaccines Global Access Facility (COVAX) [2]. As of May 2025, only 33% of the population in sub-Saharan Africa was fully vaccinated, and booster coverage stood at just 6% [3]. The persistent gap in immunization—especially among high-risk occupational groups—highlights the urgent need for localized evidence to inform health workforce protection strategies in resource-constrained health systems.

Post-vaccination SARS-CoV-2 infections—often referred to as breakthrough infections *–* have become increasingly common, particularly with the emergence of immune-evasive variants. Recent studies estimate the incidence of post-vaccination infections to range between 0.4 and 101.7 cases per 100 person-years, depending on the variant, vaccine type, and population studied, with higher rates observed during the Omicron wave [4–7]. Though these infections are typically milder in vaccinated individuals such cases [5,6,8–12], their occurrence reveals critical gaps in protection especially in populations without access to boosters or vaccine diversity.

The ChAdOx1nCoV-19 (Oxford–AstraZeneca COVISHEILD) vaccine, a deoxyribonucleic acid-based viral vector vaccine, has been widely used in LMICs. This vaccine, developed by Oxford University and AstraZeneca, consists of a non-replicating chimpanzee adenovirus that carries DNA encoding (instructions) for the spike protein of SARS-CoV-2 into the human cells [13]. The DNA is released into the cytoplasm and then to the nucleus, where it is transcribed into mRNA and translated into spike protein, eliciting an immunogenic response. This response includes the production of neutralising antibodies and activation of T-cells, which are crucial in recognising and neutralising the SARS-CoV-2 virus upon subsequent exposure [13]. While this vaccine has proven effective in reducing the risk severe disease, hospitalization, and death [14–17], its protection wanes over time and is significantly reduced against newer variants such as Omicron, especially in the absence of booster doses [18].

Healthcare workers in LMICs are among the most exposed occupational groups due to their direct, prolonged contact with patients and potentially contaminated environments [19]. Their elevated infection risk stems from both hospital-associated and community-acquired exposure [20,21]. Early in the pandemic, studies showed that hospital-associated transmission was the main primary route of infection [22,23], although community-acquired infections also played a significant role, particularly during periods of widespread transmission [20,24,25]. Hospital-associated infections often result from healthcare worker-to-healthcare worker transmission rather than patient-to-healthcare worker transmission, with a cross-sectional study estimating 90.9% of such infections originating within hospitals [20].

Globally, the prevalence of SARS-CoV-2 infection among healthcare workers is estimated at 11.0% [26], with sub-Saharan African countries reporting rates as high as 59.3% [27]. In Ghana, the healthcare workers case fatality rate stands at 1.7%, with infection rates reaching some of the highest levels across the region. The WHO estimated that between January 2020 and May 2021, the number of COVID-19-related deaths among healthcare workers was between 80,000 and 180,000 [28]. In response, many healthcare institutions implemented infection prevention and control (IPC) strategies, including vaccine prioritization and expanded booster access [29]. However, these interventions have been constrained by limited booster uptake, continued reliance on a single vaccine platform, and persistent vaccine hesitancy which is driven by safety concerns, misinformation, and low trust in heterologous booster combinations. Protecting healthcare workers remains a strategic imperative—not only to safeguard their health but also to preserve essential healthcare system functionality during epidemic surges.

In Ghana, the COVID-19 vaccination campaign started on March 3^rd^, 2021, with ChAdOx1nCoV-19 (Oxford-AstraZeneca COVISHEILD) vaccine being the only licensed vaccine available at the time. Healthcare workers were prioritized for early vaccination due to their elevated exposure risk. As of 31 December 2023, approximately 13.8 million adults in Ghana had received at least one dose of a COVID-19 vaccine, and 10.7 million were fully vaccinated, out of a total national population of approximately 33 million [30,31]. However, booster coverage remains critically low, and the country’s reliance on a single vaccine platform during the early phase left little room for flexibility in responding to variant-driven waves [2].

With the continued workplace exposure risk among healthcare workers and limited booster uptake, we conducted a cohort study to investigate the incidence and risk factors associated with post-vaccination SARS-CoV-2 infections among healthcare workers at a tertiary hospital in Ghana following the administration of the ChAdOx1 nCoV-19 vaccine. The goal is to generate context-specific evidence to inform booster prioritization, enhance occupational protection, and guide pandemic response in resource-constrained health systems.

## Method and material

### Study population and recruitment

This observational cohort study was conducted at the Korle-bu Teaching Hospital, Ghana’s premier hospital, with a bed capacity of 2000. After obtaining ethical approval, the study was publicised through hospital noticeboards and social media platforms. All hospital staff were sensitised about the COVID-19 cohort study through these channels and departmental presentations. Eligible healthcare workers were invited to participate in the study after indicating their willingness to do so by preregistering with their departmental public health nurses or through an online platform.

To be eligible for the study, a healthcare worker had to be 18 years old and above, not ill, tested negative for both antigen and PCR tests, and had either been fully (two doses) or partially (one dose) vaccinated with ChAdOx1nCoV-19 vaccine. The vaccine doses and the type of vaccine received were confirmed by entering participants’ vaccination card numbers into the Ghana national vaccination database.

### Ethical approval

This research was conducted in accordance with the Declaration of Helsinki. The Institutional Review Board of the Korle-bu Teaching Hospital (KBTH-ADM/00014/2021) approved the study protocol before the beginning of the research activities. Details of the study and symptom monitoring surveillance were explained to each healthcare worker before written informed consent was obtained. All participant information was kept confidential and used only for study purposes.

### Inclusivity in global research

Additional information regarding the ethical, cultural, and scientific considerations specific to inclusivity in global research is included in the Supporting Information (S1 File).

### Data collection at baseline

Study data were collected and managed using REDCap (Research Electronic Data Capture) [32,33], a secure, web-based software platform designed to support data capture for research studies. The REDCap system was hosted and managed by Korle-bu Teaching Hospital, with support from the hospital’s Biostatistics Team for development and maintenance. If participants were unable to complete the survey on their phone or tablet, the survey link was sent via REDCap for completion by email, or a trained research assistant called to obtain responses by phone.

A self-administered baseline questionnaire was used to obtain information about the variables of interest, which included demographic information, occupation, vaccination status, clinical stations, frontline status and clinical information on hospitalisation and death. Healthcare workers’ previous SARS-CoV-2 infections were confirmed by the hospital’s polymerase chain reaction database for COVID-19 infections.

### Data security and handling, confidentiality, and privacy

All data and research records were stored on secure, firewall-protected, and password-protected REDCap platforms hosted on the hospital’s secure server. Access to the REDCap platform was restricted to authorised users with individual usernames and passwords, enabling data entry and tracking of study participants. At the completion of the study, the REDCap administrator provided de-identified data to the investigators for analysis. Participant confidentiality was maintained throughout the study by adhering strictly to the Korle-bu Institutional Review Board guidelines and Ghana’s medical record policies.

### Vaccination

All study participants were vaccinated with ChAdOx1nCoV-19 (AstraZeneca COVISHIELD). Details of vaccine administration have been documented elsewhere [34].

### Ascertainment of the primary outcome

The primary outcome of post-vaccination infection was defined as time to first positive real-time reverse transcription polymerase chain reaction (rt-PCR) test at least 21 days after the first-dose vaccination in the partially vaccinated cohort or seven days after the second-dose vaccination in the fully vaccinated cohort.

Korle-bu Teaching Hospital adopted a specific policy on Wednesday, 3rd March 2021, to reduce SARS-CoV-2 infection among healthcare workers employed in the hospital. All healthcare workers were routinely screened for SARS-CoV-2 infection at least once every month using a nasopharyngeal or nasal COVID-19 Antigen Rapid Test Device (Roche-SD Biosensor; RSDB-RAT) with sensitivity ranging from 84.9% to 87.9% and specificity ranging from 98.5% to 99.5% [35,36]. In addition, COVID-19 tests were performed for study participants based on reported symptoms through online daily symptom monitoring surveillance, contact with an infected person, contact tracing, or a request from healthcare workers and public health physicians.

Healthcare workers were asked to complete the daily online symptom monitoring surveillance and immediately report any illness or symptoms to the public health physician team so that their nasopharyngeal sample could be taken for antigen and real-time polymerase chain (rt-PCR) tests. For the SARS-Cov-2 rt-PCR, the nasopharyngeal swab samples were collected at a designated site within the Public Health Department and transported in a universal media to the Public Health Reference Laboratory for RNA extraction and subsequently reversed transcription to cDNA and amplification.

All those who tested positive in less than 21 days after the administration of the first dose or less than seven days after the administration of the second dose were not considered as post-vaccination infections. All reports of Positive SARS-CoV-2 by antigen test were further confirmed by real-time polymerase chain reaction (rt-PCR). Healthcare workers who tested positive for SARS-CoV-2 were included in the active surveillance and were followed up by the public health team until they tested negative, usually within 7–14 days, according to the hospital’s policy. For confirmed positive cases, further clinical examinations were undertaken to rule out the presence of complications.

### Covariates

The online survey was used to collect baseline information, including demographic information (sex (1 = male, 0 = female), age, marital status (1 = single, 2 = married, and 3 = others (divorce, separated, and cohabitation) and religion (1 Christian, 2 = Muslim and 3 = others), previous SARS-CoV-2 infection (1 = Yes, 0 = No), vaccination status (1 = Fully vaccinated, 0 = partially vaccinated), occupation (1 = Doctors, 2 = Nurse, and 3 = Non-Cliical staff), co-morbidities (1 = Yes, 0 = No), Frontline (1 = Yes, 0 = No), and clinical station (1 = Ward, 2 = Outpatient Clinic and 3 = Emergency room). The vaccination status of the healthcare workers was classified into partially and fully vaccinated. A healthcare worker was considered partially vaccinated when they had taken only a single dose of the ChAdOx1nCoV-19 vaccine. Fully vaccinated workers were healthcare workers who completed or had taken two doses of ChAdOx1nCoV-19 vaccine. The occupations of the healthcare workers were grouped based on their primary duties: nurse, doctor, and non-clinical staff. Non-clinical staff consisted of pharmacist, laboratory technicians, orderlies, administrators, medical records, etc. Comorbidities were classified into “Yes” and “No”; “Yes” represented the healthcare workers who have Diabetes Mellitus, hypertension, asthma, sickle cell disease, liver disease, and heart disease and “No” represented those with no preexisting medical conditions.

Information on previous SARS-CoV-2 infections was retrieved from the hospital and national databases. This was done after participants provided written informed consent, allowing access to their past SARS-CoV-2 testing results for research purposes. The matching process used staff identification numbers and date of birth to accurately link healthcare workers to their corresponding infection records. This process was conducted by authorized members of the research team, in collaboration with public health data management personnel. During data retrieval, identifiable information was accessible only to designated personnel involved in the matching process. To protect privacy, all datasets were de-identified prior to analysis, and no identifiable information was shared beyond the authorized research team members involved in data linkage.

### Symptoms monitoring

Participants were monitored for the presence of 22 or more potential COVID-19-related symptoms. This included headache, fever or a history of fever, cough, runny nose or cold, muscle pain, joint pain, chest pain, dyspnea, anorexia, anosmia (loss of smell), sore throat, vomiting, throat irritation, and others.

### Follow-up

Participants were followed from the date of enrollment until the occurrence of post-vaccination SARS-CoV-2 infection, loss to follow-up, or the end of the study period. Those who did not experience infection were censored at their last recorded contact or the end of the observation period.

### Statistical analysis

All analyses were performed using R software, version 4.3.0 [37]. Descriptive statistics included mean and standard deviation, and percentage. The epidemic curve was plotted to visualise the distribution of post-vaccination infections over time.

The incidence of post-vaccination infection per 100 person-years of follow-up was calculated by dividing the total number of cases by the total person-years for all healthcare workers. We used the Kaplan-Meier method to estimate the cumulative incidence of the occupation groups and vaccination status.

The Cox proportion hazard model was used to estimate the hazard rate of factors associated with post-vaccination SARS-CoV-2 infections. First, a univariate Cox regression model was fitted for each covariate. Covariates associated with the risk of post-vaccination infections with a p-value less than 0.25 were included in the final multivariable Cox regression, adjusting for other variables. This iterative process for variable selection is known as “purposeful variable selection” [38]. A proportional hazard assumption was tested by using the Schoenfeld residuals, which showed no significant deviation over the study duration, indicating that the model’s assumptions were met.

## Results

### Study participants

**Fig 1:**
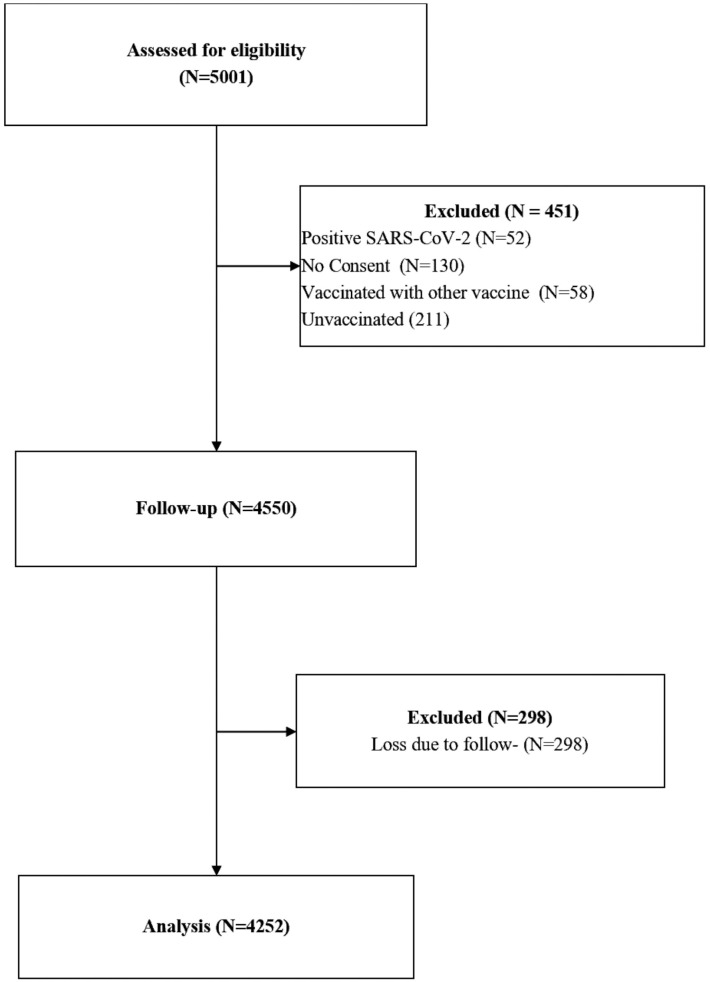
Participant flow in the study. Out of 5001 healthcare workers invited to participate, 451 were excluded due to positive SARS-CoV-2 status, lack of consent, being unvaccinated, or having received a vaccine other than ChAdOx1nCoV-19. A total of 4550 (90.1%) participants were followed for one year. After excluding 298 participants, 4252 (85.0%) were included in the final analysis.

A total of 5,001 healthcare workers were recruited from various hospital departments between May 1, 2021, and June 5, 2021. Fig 1 presents the flow of participants in the study. Of these, 451 were excluded: 52 had tested positive for SARS-CoV-2 prior to enrollment, 130 declined to provide consent, 211 were unvaccinated, and 56 had received COVID-19 vaccines other than ChAdOx1 nCoV-19, such as Moderna, Pfizer-BioNTech, or Johnson & Johnson. All the 56 individuals had received their vaccination outside Ghana, and their vaccination status could not be independently verified through the national vaccination database.

A total of 4,550 eligible healthcare workers (90.1%) were enrolled and followed from June 15, 2021, to June 15, 2022. Of these, 6.0% had no follow-up information due to incomplete symptom monitoring, missing contact details, or loss to follow-up. The remaining 4,252 healthcare workers were followed for post-vaccination SARS-CoV-2 infections for an average of 6.8 months. The age at the baseline ranged from 18 years to 60 years, with an average age of 40.2 years (SD = 14.6 years). Table 1 provides descriptive statistics of the cohort of healthcare workers at baseline. Most of the healthcare workers were female (60%), of the Christian faith (76.4%), fully vaccinated (76.0%), and nurses (61.4%). Half of the healthcare workers worked in the wards, and the other half worked at the point-of-entry service (outpatient clinic and emergency). A quarter (24.9%) of the healthcare workers were frontline workers. Only a few healthcare workers had previously been infected with SARS-CoV-2, and 14.8% of healthcare workers had preexisting medical conditions. The most reported preexisting medical condition was hypertension (7.6%), followed by diabetes (2.2%).

**Table 1:** Mean (SD) or percentage (number) with selected characteristics at the baseline.

Characteristics	N = 4252
**Age**	40.2 (14.6)
**Sex**	
Female	2563(60.3%)
Male	1689 (39.7%)
**Marital status**	
Single	1904 (44.8%)
Married	1704 (40.1%)
Others	644 (15.1%)
**Religion**	
Christians	3247 (76.4%)
Muslim	796 (18.7)
Others	209 (4.9%)
**Previous SARS-CoV-2**	
No	3431 (80.7%)
Yes	821 (19.3%)
**Vaccination status on date of test**	
Partially Vaccinated	1021 (24.0%)
Fully Vaccinated	3231 (76.0%)
**Occupation**	
Doctor	869 (20.4%)
Nurse	2609(61.4%)
Non Clinical	774 (18.2%)
**Station**	
Ward	2114 (49.7)
Out Patient Clinic	1304 (30.7)
Emergency Room	(834 (19.6)
**Frontline workers**	
No	3193 (75.1%)
Yes	1059 (24.9%)
**Pre-exiting Medical Condition**	
No	3623 (85.2%)
Yes	629 (14.8%)
**Hypertension**	
No	3930 (92.4%)
Yes	322 (7.6%)
**Diabetes**	
No	4153 (97.7%)
Yes	99 (2.2%)

### Epidemic curve for post-vaccination infections

**Fig 2:**
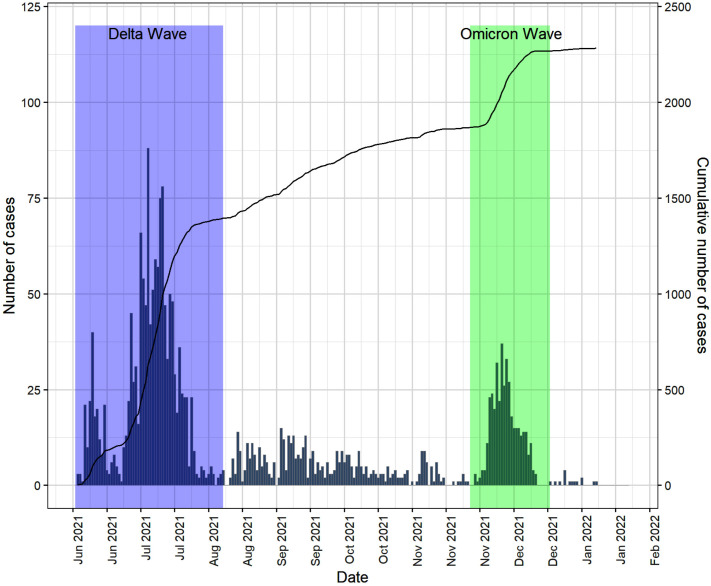
Epidemic and cumulative curves. Two epidemic waves were observed during the study: the Delta wave (June to August 2021) and the Omicron wave (December 2021). Forty percent of post-vaccination infections occurred during the Delta wave, and 30% during the Omicron wave. The total cumulative number of infections was 2283.

The Epidemic curve (Fig 2) shows two major waves of post-vaccination infections. The first wave corresponding to the Delta variant, spanned from 20^th^ June 2021–17^th^ August 2021, while the second wave, associated with the Omicron variant, occurred between 7^th^ December 2021–25^th^ December 2021. The Delta wave had the largest epidemic size accounting for about half (47.5%) of the post-vaccination infection compared to 30.0% during the Omicron wave.

### Presenting symptoms of post-vaccination infections

**Fig 3:**
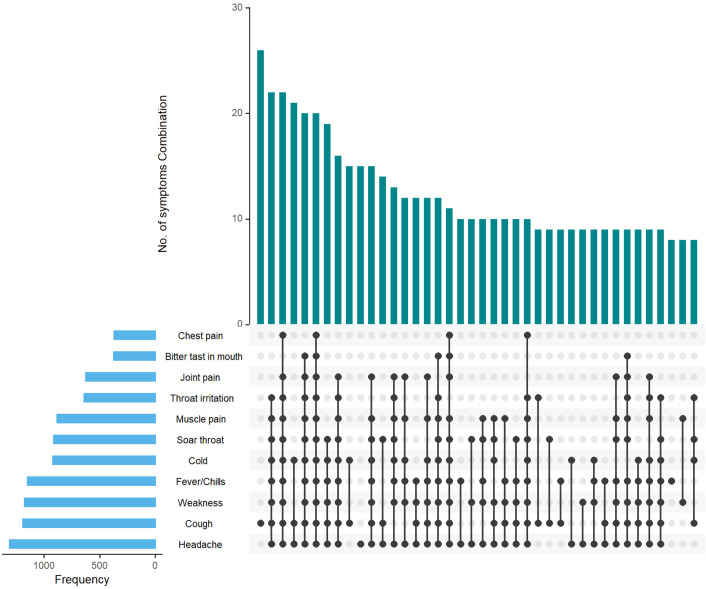
Symptom profile of post-vaccination infections. The most common symptoms reported were headache, cough, generalized weakness, fever or a history of fever, and cold. The combination of headache, fever, cold, sore throat, muscle pain, and throat irritation was the most frequent symptom grouping. Cough was the most reported single symptom.

Among 2283 infected healthcare workers, 12.4% (283) were asymptomatic. Fig 3 shows the various clinical symptoms and symptom combinations observed, with headache, cough, generalised weakness, fever or history of fever, and cold being the top presenting symptoms. The most common symptom combinations were headache, cough, generalised weakness, fever or history of fever, cold, sore throat, muscle pain and throat irritation, consistent with coryza symptoms. Most of the healthcare workers had mild post-vaccination infection with nine hospitalisations, one admitted to the intensive care unit, and zero deaths.

### Incidence rate of post-vaccination infection

The overall incidence of post-vaccination infections among healthcare workers was 95.7 cases per 100 person-years (95% CI: 91.8–99.7). Healthcare workers who were partially vaccinated had a higher incidence rate of 156.4 cases per 100 person-years (95% CI: 144.9–168.6) compared to 82.0 cases per 100 person-years (95% CI: 78.0–86.0) in those fully vaccinated.

Clinical healthcare workers, particularly nurses and doctors, had a higher incidence of post-vaccination infections than non-clinical healthcare workers. Nurses reported a high incidence of post-vaccination infection, followed by doctors and then non-clinical healthcare workers.

**Fig 4:**
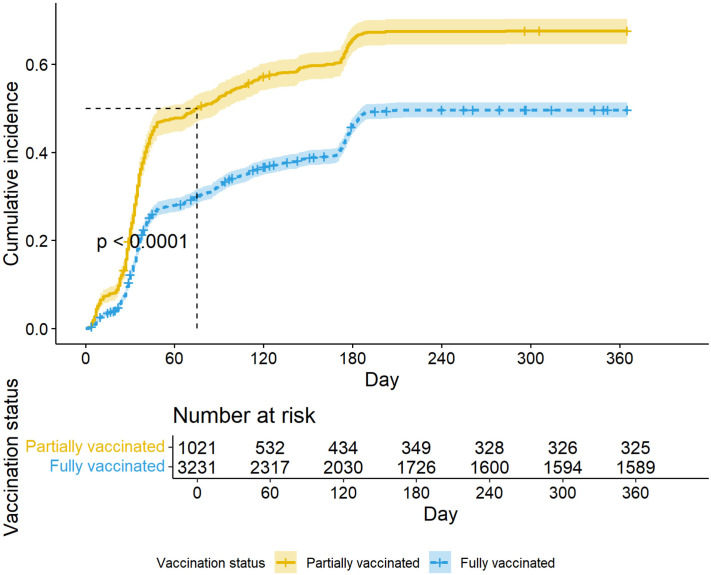
Cumulative incidence of post-vaccination infections by full and partial vaccination status. The cumulative incidence of post-vaccination infections was significantly higher in participants who were partially vaccinated compared to those fully vaccinated (p < 0.00).

**Fig 5:**
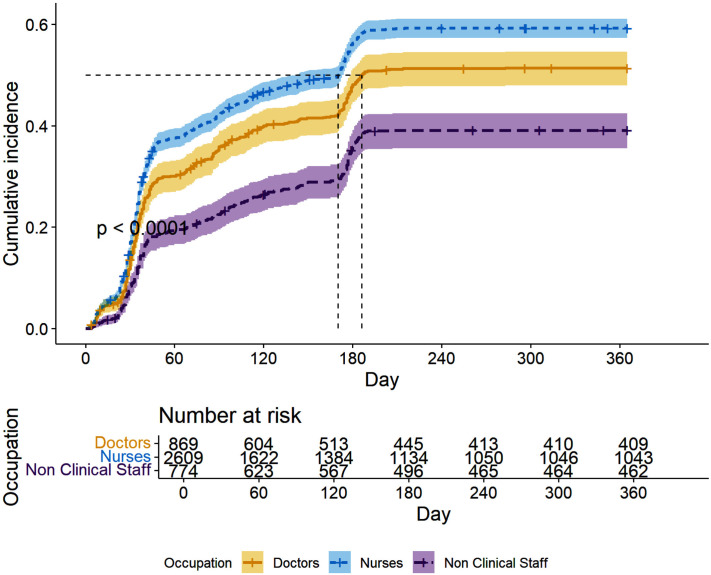
Cumulative incidence of post-vaccination infections by occupation. Cumulative incidence curves showed significant differences in post-vaccination infection rates by occupation. Nurses had the highest cumulative incidence, followed by doctors, with non-clinical healthcare workers having the lowest incidence (p < 0.00).

The cumulative incidence of post-vaccination infection in 3, 6, and 12 months were 37.4% (95% CI: 35.9% − 38.8%), 50.9% (95% CI: 49.4% − 52.4%), and 53.9% (95% CI 52.5% −55.5%), respectively with partially vaccinated workers experiencing a higher cumulative incidence across the study duration (Fig 4). Additionally, the cumulative incidence of the nurses and doctors was higher than that of non-clinical healthcare workers (Fig 5).

### Factors associated with post-vaccination infections

Table 2 summarises results from multivariate analysis that established an association between post-vaccination infections and covariates using Cox regression. Healthcare workers who were fully vaccinated had a 58.0% reduction in risk of post-vaccination infections compared with those who were partially vaccinated after adjusting for other covariates. Comparing clinical and non-clinical healthcare workers, nurses experienced an almost two-fold increase in the rate of post-vaccination infections compared with non-clinical healthcare workers. Similarly, doctors had a 64.0% increase in the risk of post-vaccination infections compared with non-clinical healthcare workers.

**Table 2:** Crude and adjusted HRs for post-vaccination SARS-CoV-2 infections.

Characteristics	Post-vaccination infection		Univariate analysis		Multivariate analysis	
	Yes N(%)	No N(%)	Hazard Ratio (95% CI)	P value	Adjusted Hazard Ratio (95% CI)	P value
Age* (per 5 years)	8.2 (3.1)	7.7 (2.7)	1.06 (1.04, 1.07)	<0.01	1.04 (1.02,1.05)	<0.01
**Sex**						
Female	1394 (54.4)	1169(45.6)	1		1	
Male	889 (52.6)	800 (47.6)	0.96 (0.88,1.04)	0.33	0.95 (0.87,1.03)	0.23
**Marital status**						
Single	1014 (53.3)	890 (46.7)	1		1	
Married	930 (54.6)	774 (45.4)	1.04 (0.95, 1.14)	0.38	1.06 (0.97, 1.16)	0.22
Others	339 (52.6)	305 (47.4)	0.99 (0.87, 1.12)	0.85	1.04 (0.92, 1.18)	0.51
**Religion**						
Christians	1489 (54.2)	1258 (45.8)	1		1	
Muslim	576 (52.6)	520 (47.4)	0.96 (0.88, 1.06)	0.45	0.94 (0.85, 1.04)	0.32
Others	218 (53.3)	191 (46.7)	0.97 (0.84, 1.12)	0.68	0.95 (0.83, 1.10)	0.63
**Previous SARS-CoV-2**						
No	1916 (55.8)	1515 (44.2)	1		1	
Yes	367 (44.7)	454 (55.3)	0.75 (0.64,0.80)	<0.01	0.79 (0.70,0.88)	<0.01
**Vaccination status on date of test**						
Partially Vaccinated	688 (67.4)	333 (32.6)	1		1	
Fully Vaccinated	1595 (49.4)	1636 (50.6)	0.58(0.53,0.63)	<0.01	0.56 (0.51,0.62)	< 0.01
**Occupation**						
Non Clinical	301 (38.9)	473 (61.1)	1		1	
Doctor	442 (50.9)	427 (49.1)	1.49 (1.29,1.72)	<0.01	1.45 (1.29,1.73)	<0.01
Nurse	1540 (59.9)	1069(41.0)	1.87 (1.65,2.11)	<0.01	1.91 (1.69,2.17)	<0.01
**Station**						
Ward	982 (46.5)	1132 (53.5)	1		1	
Out Patient Clinic	735 (56.4)	569 (43.6)	1.33 (1.21, 1.46)	<0.01	1.37 (1.24, 1.51)	<0.01
Emergency Room	566 (67.9)	268 (32.1)	1.87(1.68, 2.07)	<0.01	2.05 (1.85, 2.28)	<0.01
**Frontline workers**						
No	1528 (47.9)	1665 (52.1)	1		1	
Yes	755 (71.3)	304 (28.7)	2.01 (1.84, 2.19)	<0.01	2.15 (1.97, 2.35)	<0.01
**Pre-existing Medical Condition**						
No	1831 (50.5)	1792 (49.5)	1		1	
Yes	452 (71.9)	177 (28.1)	1.86 (1.68,2.06)	<0.01	1.86 (1.67,1.73)	<0.01

* Presented in mean and standard deviation

Frontline healthcare workers at the baseline were at a significantly higher risk of post-vaccination infections compared with non-frontline healthcare workers. This effect was consistent across the different entry points of services; the hazard ratio (95% CI) for healthcare workers in the emergency rooms was 1.37 (95% CI: 1.24–1.51), and for outpatient clinics was 2.05 (1.87–2.28).

In general, the rate of post-vaccination infections increased by 5% with every five (5) years increase in age. This indicates that elderly healthcare workers were more susceptible to post-vaccination infections compared to a younger group of healthcare workers. Healthcare workers who previously had SARS-CoV-2 infections had a 21.0% decrease in infection rate compared to those with no previous infection. Healthcare workers with pre-existing conditions were at higher risk of post-vaccination infections compared with those with no pre-existing conditions.

## Discussion

As vaccination remains the best and most powerful primary prevention strategy, it has become imperative to understand the incidence and risk factors associated with post-vaccination SARS-CoV-2 infections among healthcare workers in Ghana. These findings provide critical insight for LMIC policymakers navigating booster rollout and healthcare worker protection strategies in resource-constrained environments.

This study followed a cohort of healthcare workers vaccinated with the ChAdOx1nCoV-19 vaccine over one year. Overall, 2283 post-vaccination infections were reported among 4252 healthcare workers after one year of follow-up. This incidence is higher than that reported in other studies among healthcare workers due to underreporting of cases, as many vaccinated people usually experience no (asymptomatic) or mild symptoms and are less proactive in seeking COVID-19 testing [9,29,39]. However, our study confirms the high incidence of post-vaccination infection among healthcare workers found in similar studies [40,41].

Full vaccination with ChAdOx1nCoV-19 vaccine offered more protection against infection than partial vaccination [29,41]. It is unclear why some healthcare workers remained partially vaccinated despite the availability of the vaccine by the Ministry of Health. Possible reasons for taking only a single dose of the ChAdOx1nCoV-19 vaccine include adverse events following the first dose, concerns about vaccine efficacy, superstitions, and other personal or systemic factors. Additionally, some healthcare workers expressed reluctance to receive a heterologous booster due to safety concerns, and strong preference for the initial vaccine platform. These beliefs, despite updates in national policy, have continued to negatively influence booster uptake and reflect broader patterns of vaccine hesitancy in LMICs. Several studies confirm that fear of adverse effects, misinformation, and mistrust of new vaccine platforms have deterred many healthcare workers from accepting heterologous boosters, even where supply was adequate [42–45].

This vaccine hesitancy was further reinforced by Ghana’s early reliance on a single vaccine platform (ChAdOx1 nCoV-19), a strategy shaped by global vaccine inequity and delayed access to alternative vaccines. Due to limited supply and distribution constraints, healthcare workers had few choices, and the lack of timely access to booster options hindered public confidence in mixed-platform strategies. Studies have shown that such single-platform dependency often as a consequence of vaccine inequity, especially when not accompanied by heterologous booster acceptance policies, undermines full immunization in LMIC settings [46,47].

In Ghana, the Ministry of Health recommends booster doses for healthcare workers and other vulnerable groups [48–50]. Delivery has been via periodic campaigns and integration into primary care rather than inclusion in the childhood Expanded Programme on Immunisation (EPI) schedule [51]. During our study period, vaccine supply in Ghana depended largely on COVAX allocations and partner support, with intermittent shortages typical of many LMICs, which likely affected the availability and uptake of boosters among healthcare workers. In July 2025, following a resurgence of COVID-19 cases, the government announced renewed procurement measures (including plans for in-country sourcing) to secure vaccine supply [52]. These post-period actions did not affect our estimates but illustrate how policy and supply dynamics can influence booster availability and uptake among healthcare workers.

Our findings suggest that improving booster uptake will require proactive education campaigns that emphasize the safety and efficacy of mixed-vaccine platforms. Such efforts are especially critical in LMICs where delays in booster supply threaten the protection of high-risk populations such healthcare workers. Policymakers should integrate these campaigns into broader health workforce safety strategies

Our results show a differential post-vaccination infection risk among healthcare workers. Clinical healthcare workers, particularly nurses, had a higher risk of infection than nonclinical staff. Nurses were more vulnerable than doctors due to their work in high-risk settings, which often involved close contact and hands-on care. These findings are consistent with other studies conducted among healthcare workers in different countries [29,40,53]. The high number of infections among clinical healthcare workers can be attributed to multiple factors, including prolonged and close contact with infected patients and working in a high-pressure environment where maintaining infection control is difficult [29,40,41,53–55]. However, this elevated risk can be linked to SARS-CoV-2 infections in the community [56]. During periods of high community transmission, the background risk of infection increases, which can contribute to increased patient-healthcare workers transmission, particularly those in outpatient clinics. Outpatient clinics may carry a higher infection risk than wards due to high patient turnover, incomplete triaging of symptomatic patients, and shorter visits, which limit opportunities to enforce infection control measures [57]. Nurses working in outpatient settings may be more exposed to asymptomatic or undiagnosed cases, compounding their risk.

Additionally, our results confirmed previous studies that healthcare workers in frontlines and point-of-entry roles, such as emergency rooms and outpatient clinics, were at a higher risk of infections [58,59]. Healthcare workers in fast-paced and high-volume clinical settings have less time to implement optimal infection prevention and control (IPC) measures. For example, patients with undiagnosed infection may inadvertently transmit the virus to all attending healthcare workers, indicating the volatility of the hospital environment during an outbreak.

Our findings also highlight the impact of epidemic dynamics on post-vaccination infection risks among healthcare workers. The Delta wave in Ghana was associated with a larger epidemic size compared to the Omicron wave, despite the latter being more transmissible [60,61]. This can be attributed to differences in vaccine coverage and testing patterns during the respective waves. The Delta wave coincided with lower vaccine coverage and higher rates of severe disease, which likely led to increased testing uptake among healthcare workers [62,63]. In contrast, the Omicron wave occurred during a period of higher vaccine coverage, and many cases presented with milder symptoms, potentially reducing the perceived need for testing [62]. Additionally, the rapid spread of Omicron may have saturated the susceptible population more quickly, leading to a shorter wave duration [61]. These findings show how vaccination, disease severity, and testing patterns together influence infection trends among healthcare workers, and remain especially important for LMICs where booster coverage is still very low.

In our multivariable analysis, age, comorbidities, and previous SARS-CoV-2 infection were independent predictors of post-vaccination infection among healthcare workers. From our results, we confirmed the widely reported finding that elderly healthcare workers, especially those partially vaccinated, were more vulnerable to post-vaccination infection [64,65]. This vulnerability is because of the inability of elderly individuals to mount an effective immune response to vaccination, limiting vaccination efficacy [66–68]. Thus, our findings reinforce need for prioritizing booster campaigns for elderly healthcare workers.

The effect of sex on post-vaccination infections is unclear. Some studies found men at higher risk [69,70], while others have found women to be at higher risk [71–73]. Additionally, some studies [9,74], like our study, observed no significant difference between male and female healthcare workers, despite the increased number of infections among women, reflecting nurses as the predominant profession in the hospital.

Among healthcare workers, previous SARS-CoV-2 infection was associated with a reduced risk of post-vaccination infection. Our results concur with other studies that prior infection enhances the immune response to vaccination. One study showed strong production of neutralising antibodies after first-dose vaccination in people with previous SARS-CoV-2 infection, which did not increase further following the second dose [75].

Comorbidities are known to be associated with SARS-CoV-2 in unvaccinated populations [65,76,77]. Our results suggest that healthcare workers with comorbidities such as hypertension and diabetes were at higher risk of post-vaccination infection, supporting previous findings that individuals with comorbidities have a reduced spike-specific SARS-CoV-2 immune response in both cellular and serological compartments of the adaptive immune system [68].

The increase in post-vaccination infections during the epidemic waves emphasizes the need for additional doses to reinforce prior vaccine-acquired immunity among healthcare workers, especially those in high-exposure roles and vulnerable groups such emergency workers, elderly workers, and those with comorbidities. In addition, hospital administrators need to implement enhanced risk-reduction strategies -including regular personal protective equipment (PPE) audits, optimized ventilation in high-traffic and enclosed areas, and infection prevention and control training during outbreaks – to protect healthcare workers and sustain service delivery. These strategies should be embedded in institutional policy and crisis preparedness planning.

Our prospective study had the advantage of collecting information on possible risk factors before the post-vaccination infection, eliminating the possibility of recall bias once post-vaccination infection has occurred. All study participants were recruited from one tertiary hospital, eliminating the hospital as a potential source of variability. With a sample size of 4252 healthcare workers, this study has high statistical power to detect subtle effects in incidence.

However, this study has several limitations. The use of a SARS-CoV-2 antigen test kit as the primary screening tool, with confirmation of positives using RT-PCR, carries the risk of false negatives, potentially underestimating the true number of infections. We could not perform genomic sequencing on every SARS-CoV-2 sample to determine its variant. During the epidemic wave, genomic sequencing was performed in only 30% of the initial positive samples to identify variant of the concern. The study could not account for all possible potential confounders (residual confounders). For example, changes in the personal protective equipment used and other mitigating factors were not accounted for, which can affect the infection rate. The waning effect of the vaccine-induced immunity has been observed [75,78,79], so the extent to which this increases the risk of post-vaccination infection was not considered in this study. In addition, No tests were conducted to confirm whether vaccinated healthcare workers were fully protected, as some studies have shown that not all vaccinated individuals mount an effective immune response [66,68,75,78].

## Conclusion

In conclusion, the results of our study indicate a high incidence of post-vaccination infection among healthcare workers, with an elevated risk observed in partially vaccinated individuals, older staff members, those with comorbidities, and nurses in high-contact roles. These findings support the need for tailored booster vaccination strategies and strengthened infection control, particularly in resource-limited settings.

The results also highlight how early reliance on a single vaccine platform and hesitancy toward heterologous boosters may have limited protective coverage. Addressing these gaps requires flexible vaccination policies, proactive communication on vaccine safety, and sustained support for the protection of healthcare workers in LMICs. Future research should explore strategies to improve booster uptake and optimise protection for high-risk groups.

## Supporting information

S1 File Inclusivity in global research questionnaire.
(DOCX)
